# What's in a name? Role of verbal context in touch

**DOI:** 10.1098/rsos.221147

**Published:** 2022-11-30

**Authors:** Supreet Saluja, Karina Chan, Tully Lynch, Richard J. Stevenson

**Affiliations:** Department of Psychology, Macquarie University, Sydney, NSW 2109, Australia

**Keywords:** verbal context, tactile behaviour, affective perception, touch

## Abstract

Can a name (i.e. verbal context) change how we react to and perceive an object? This question has been addressed several times for chemosensory objects, but appears unanswered for touch. To address this, two studies were run. In each, we allocated participants to a Positive, Neutral or Negative Group, and asked them to touch the same four objects, twice—first, named by the experimenter according to their Group-name, and second, named by the participant. Participants were timed as they touched and rated the objects on pleasantness and disgust. Negative-named objects were touched for shorter durations, and rated more negatively, than neutral-named objects, and positive-named objects were touched for the longest and rated most positively. In the second presentation, most objects (greater than 90%) were named by participants in accordance with their assigned Group-names. The similarity of these findings to chemosensory verbal context effects and their mechanistic basis is discussed.

## 1. What's in a name? Role of verbal context in touch

Shakespeare posed the question ‘What's in a name?’ asserting that ‘A rose by any other name, would smell as sweet’. We know he was wrong for smell [1,2], and here we examine his supposition as it pertains to touch—that is, can a name alter one's reaction and perception of a touch object?

The ability of names to alter how we perceive and respond to stimuli is known as the verbal context effect [3]. Names (or labels, descriptors) only affect liking (affective perception) and sensory perception of *ambiguous* sensory stimuli, which have variant interpretations [4,5]. Thus, while some verbal context effects have been shown for vision (using abstract paintings) and audition (using vague environmental sounds), they are most extensively studied in the chemical senses, which seem more prone to ambiguity [6–8]. In taste, naming a bitter taste as mildly aversive increases its perceived pleasantness, relative to naming it highly aversive [9]. Similarly, presenting foods with false descriptors (e.g. ‘soy’ [10] or ‘organic’ [11]) or a false name (e.g. ‘ice-cream’ for salmon mousse [12]) affects not only liking but also sensory perception (i.e. its perceived bitterness, graininess and fattiness). In olfaction, participants will shift their ratings of pleasantness to be congruent with the effect (Positive, Neutral and Negative) linked to the odour's name [4,13]. For instance, isovaleric-butyric acid is perceived as pleasant when named parmesan (positive name) but unpleasant when named vomit (negative name [1]). Giving odours a negative (or disease-related) name can also increase participants' disgust to them—i.e. a basic emotion, which facilitates avoidance of diseased sensory objects [14,15]. Verbal context also alters olfactory behaviour—i.e. negative-named odours are sniffed less than positive-named ones [2]. Olfactory verbal context effects are likely so common because odours are often invisible, poorly localized to a source and difficult to verbally describe and identify [16].

In contrast with the other sensory modalities, touch has received little investigation of its susceptibility to verbal context effects. Some evidence suggests that verbal context effects in touch may occur [17–19], yet it remains unknown whether names alter behavioural and affective responses to touch objects. Consequently, we conducted two studies to test how people would respond to and perceive touch objects, when they were presented with different names. In each, participants were allocated to a Positive, Neutral or Negative Group, and asked to touch the same four objects—named according to their assigned Group-name—for as long as they wished. We measured participants' touch duration, pleasantness and disgust. We hypothesized that there would be a linear trend of Group (Negative, Neutral and Positive) on touch duration, and pleasantness and disgust ratings.

## 2. Study 1

### 2.1. Participants

Thirty-four psychology students (21 women) aged from 18 to 32 years (*M* = 19.8; s.d. = 2.0), who were healthy and reported normal tactile perception, consented to participate in a study examining touch and emotion. The Macquarie University IRB approved the study (5201952547623). Sample size was based on effect sizes derived from past verbal context studies (i.e. Cohen's *d*s ranging from 0.5 to 1, namely, large effect sizes; [17,20])—which equated to approximately 11 participants/Group.

### 2.2. Tactile stimuli

Four tactile stimuli were used and these were all presented at room temperature in the experiment. The tactile stimuli were given either positive-names (in the Positive Group), neutral names (in the Neutral Group) or negative-names (in the Negative Group; table 1). Names were piloted on perceived pleasantness (−50 ‘*very unpleasant*’ to 50 ‘*very pleasant*’) and disgust (0 ‘*not at all’* to 100 *‘very’*), on 20 participants. In the pilot-data, negative-names were rated as significantly less pleasant and more disgusting than neutral names, which, in turn were significantly less pleasant and more disgusting than positive-names (all *t*_19_'s > 5.1, *p* < 0.001).

**Table 1.** Tactile stimuli and names given for each Group (Positive, Negative and Neutral*).*

tactile stimuli	Positive name	Negative name	Neutral name
eucalyptus leaves	Native Australian eucalyptus leaves	dead moths	dried leaves
kimchi (Paldo brand)	aloe vera leaves in gel	off-lettuce	kimchi
coffee-puck (from coffee machine)	homemade chocolate cookies	horse-poo	coffee grounds
mayonnaise (kraft)	moisturizer	spoilt yoghurt	softened butter

### 2.3. Experimental procedure

**Figure 1.**
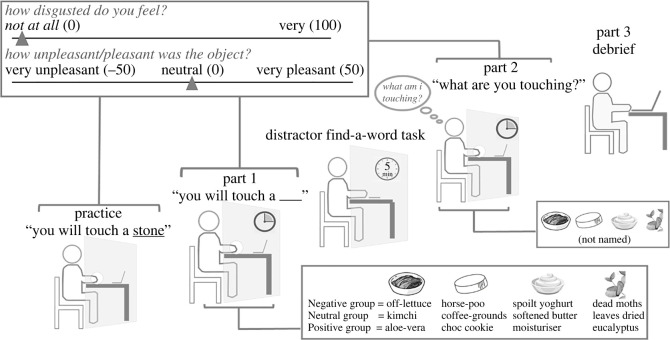
Procedure of experiment.

Participants were informed that the study looked at touch and emotion and they would have to simultaneously touch and rate objects, on various computer-based scales. Following consent, participants placed nose plugs in their nostrils to avoid olfaction from potentially cueing object identity. There were no obviously discernible auditory cues to identity. The experimenter then put disposable gloves on. The experiment began and had four parts: Practice, Part 1, Part 2, Part 3—figure 1.

#### 2.3.1. Practice

Participants placed their left arm through a screen to their left and touched a stone (practice object), which was placed underneath their hand by the experimenter. While touching the stone, participants rated it on disgust and pleasantness. Once touching ceased, the experimenter cleaned (via sanitizer wipes) and dried (via paper towels) participants' hands, which remained behind the screen for the duration of the study.

#### 2.3.2. Part 1

Participants were given four objects, named according to their Group (table 1), to simultaneously touch and rate. The onset to the offset of participant's object-touching was timed using a concealed stop watch, as the experimenter could see the participant's hand. The stopwatch was paused if touching temporarily ceased and resumed if touching recommenced. The experimenter cleaned the participant's hand in between each object. After Part 1, participants completed a 5 min distractor find-a-word task.

#### 2.3.3. Part 2

Part 2 was the same as Part 1, except in Part 2 participants were *not* given the object names, and had to write down what they thought they were touching.

#### 2.3.4. Part 3

Participants were debriefed and told the study assessed verbal context on affective perception and behaviour. Knowing the true aims, all participants reconsented.

### 2.4. Analysis approach

The study was conducted from March 2021 to August 2022, and neither of the studies reported were pre-registered. All data are accessible online from Dryad (see: https://doi.org/10.5061/dryad.zgmsbccfr). SPSS v. 26 was used for statistical analyses, with graphs prepared in R v.4.2.0. Data were collapsed across object-type in each Group. As data were normally distributed, parametric analyses were used. Three separate mixed ANOVAs with Group (Negative, Neutral and Positive) as the between subjects factor and Presentation (First, Second) as the within subjects factor, were run on touch duration data, pleasantness and disgust ratings.

### 2.5. Results

**Figure 2.**
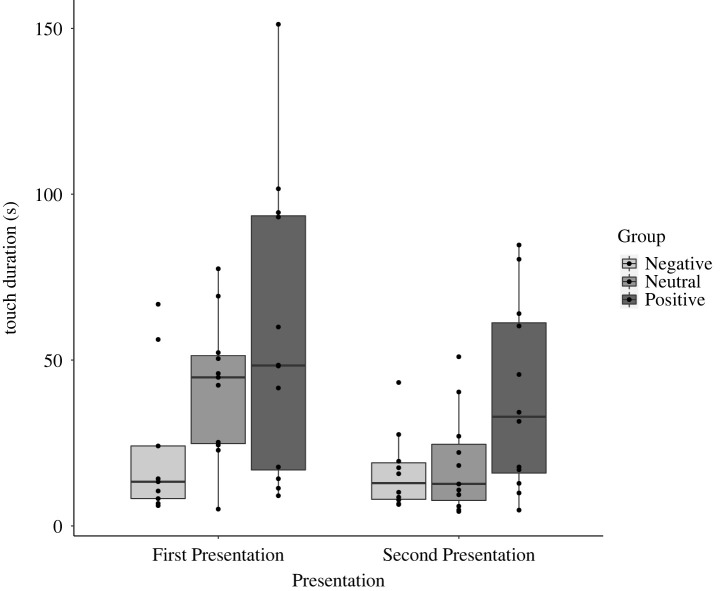
Mean touch duration for each Group (Negative, Neutral and Positive), at First and Second Presentation.

As predicted, we found a significant linear trend of Group on touch duration, *F*_1, 29_ = 6.69, MSE = 7.95, *η*^2^ = 0.19, *p* = 0.015. This indicated that participants in the Positive Group tended to touch the objects for longer than those in the Neutral Group, who in turn tended to touch the objects longer than participants in the Negative Group. There was also a main effect of Presentation, *F*_1, 29_ = 26.94, MSE = 66.04, *η*^2^ = 0.48, *p* < 0.001, with objects touched for a longer duration in their First Presentation, compared to their Second Presentation. There was no significant interaction effect (figure 2).

**Figure 3.**
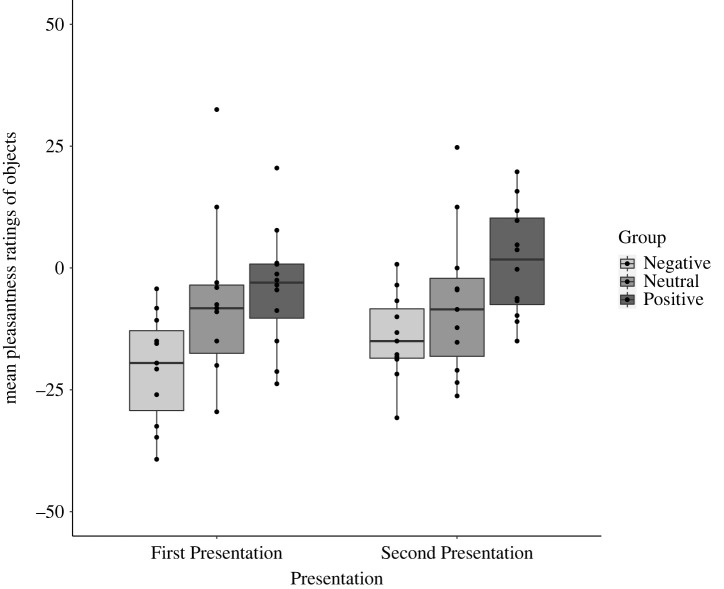
Mean pleasantness ratings for each Group (Negative, Neutral and Positive), at First and Second Presentation.

**Figure 4.**
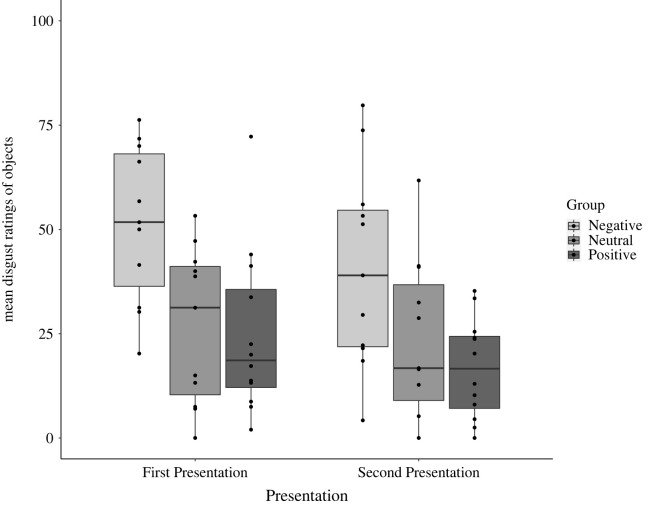
Mean disgust ratings for each Group (Negative, Neutral and Positive), at First and Second Presentation.

Also as predicted, the mixed ANOVAs revealed a significant linear trend of Group for pleasantness, *F*_1, 31_ = 9.82, MSE = 3.59, *p* = 0.004, *η*^2^ = 0.24, and for disgust ratings *F*_1, 31_ = 11.33, MSE = 5.34, *p* = 0.002, *η*^2^ = 0.27. This indicated that participants in the Positive Group tended to find the objects more pleasant and less disgusting, than those in the Neutral Group, who in turn found the objects more pleasant and less disgusting than those in the Negative Group. There was also a main effect of Presentation for pleasantness, *F*_1, 31_ = 6.40, MSE = 15.71, *p* = 0.02, *η*^2^ = 0.17, and for disgust ratings, *F*_1, 31_ = 12.37, MSE = 30.43, *p* = 0.001, *η*^2^ = 0.28. Objects were less disgusting and unpleasant during the Second Presentation, compared to their First Presentation. In both ANOVAs, there were no significant interactions (see figures 3 and 4).

Pearson correlations indicated that participants' pleasantness ratings were moderately correlated with their touch duration (*r*_33_ = 0.38, *p* = 0.03; i.e. participants touched the objects they liked more for longer), and negatively and strongly correlated to their disgust ratings (*r*_33_ = −0.80, *p* < 0.001). The correlation between disgust ratings and touch duration did not reach significance, but was in the expected direction (*r*_33_ = −0.20, *p* = 0.30). Thus, touch duration and the affective ratings likely measured the same underlying construct.

Finally, greater than 95% (130/136; i.e. 4 objects × 34 participants = 136) of the objects were given the same name in the second presentation as presented in the first, suggesting the majority of participants identified the objects with their assigned Group-name.

### 2.6. Discussion

As predicted, the name given to touch objects significantly altered how participants behaved towards and evaluated them. However, Study 1 had two limitations. First, a small sample size was used—meaning the results should be replicated. Second, a potential element of bias was present, as the experimenter was not blind to the Group allocation during the recording of the touch duration.

## 3. Study 2

Study 2 aimed to replicate Study 1, with a larger sample size, and using a blind procedure to code touch duration. To achieve this, Study 2 was identical to Study 1 except for two changes. First, a larger sample size (*N* = 51) was used. Second, each participant's hand was video recorded (unbeknownst to them) using a concealed camera, as they touched the objects. Two coders (who were blind to the study's aims and object names) then coded the videos on touch duration.

### 3.1. Participants

Fifty-one psychology students (34 women) aged from 17 to 57 years (*M* = 20.3; s.d. = 5.8), who were healthy and reported normal tactile perception, consented to participate in Study 2. Ethical approval, consent and debrief were as for Study 1, except in Study 2 participants were debriefed at the end that their hand was video recorded. All participants reconsented.

### 3.2. Experimental procedure

The experimental procedure was as for Study 1, except touch duration was recorded via a concealed video camera.

### 3.3. Coding procedure

Two coders, blind to the study's aims and object identity, coded the videos—which were muted—on touch duration. Due to video camera issues, one participant was not recorded, leading to a total of 50 videos. Five trial videos (i.e. participants touching objects unnamed, in Part 2) were timed by the experimenter and used to ensure reliability of coding. High median intraclass correlation coefficients between the experimenter and both coders in the trial videos (greater than 0.90; range 0.15 to 0.99), verified the coding procedure was reliable.

Part 1 of the videos (when the objects were named) were timed, with coder-one coding all 50 videos, and coder-two, 26/50. There was high agreement on touch duration between the coders (median intraclass correlation coefficient of 0.98; range 0.95 to 0.99), and consequently, both coders' touch duration values were averaged in the overlapping 26 videos. Part 2 was not timed to reduce coder fatigue.

### 3.4. Analyses

The analysis procedure for Study 2 was as for Study 1, except only touch duration, pleasantness and disgust ratings pertaining to Part 1 of the experiment (i.e. first presentation of objects, when objects were named) were used in Study 2 analyses. Part 2 data (when objects were presented again, unnamed) were not analysed as these data were not coded.

### 3.5. Results

**Figure 5.**
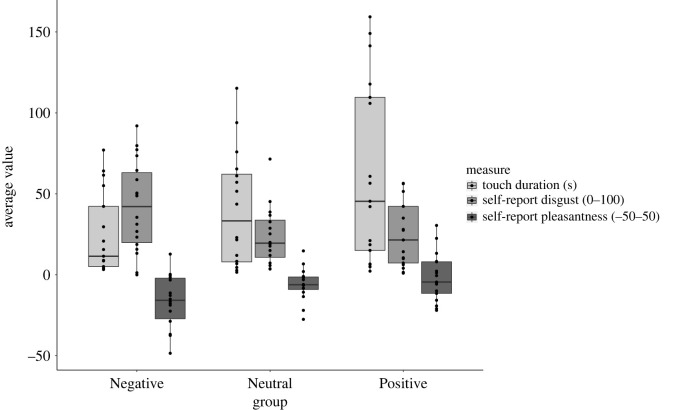
Touch duration, Pleasantness and Disgust by Group (Negative, Neutral and Positive).

As predicted, three separate one-way ANOVAs revealed a significant linear trend of Group on touch duration, *F*_1, 48_ = 7.07, MSE = 9.95, *p* = 0.011, *η*^2^ = 0.13, self-reported pleasantness, *F*_1, 49_ = 9.55, MSE = 3.38, *p* = 0.003, *η*^2^ = 0.16 and self-reported disgust, *F*_1,49_ = 4.84, MSE = 5.47, *p* = 0.033, *η*^2^ = 0.09 (figure 5). Thus, participants in the Positive Group tended to touch the objects for longer, found the objects more pleasant and less disgusting, than those in the Neutral Group, who in turn tended to touch the objects for longer, found the objects more pleasant and less disgusting than those in the Negative Group.

Paralleling Study 1, Pearson correlations indicated participants’ pleasantness ratings were moderately correlated with their touch duration (*r*_49_ = 0.40, *p* < 0.005; i.e. participants touched objects they liked for longer), and negatively and strongly correlated to their disgust ratings (*r*_50_ = −0.75, *p* < 0.001). While the correlation between disgust ratings and touch duration was in the expected direction, it did not reach significance (*r*_49_ = −0.23, *p* = 0.110). Thus, touch duration and affective ratings likely measured the same underlying construct.

Finally, more than 90% (184/205; i.e. 4 objects × 51 participants) of the objects were given the same name in the second presentation as in their first, suggesting that in Study 2, the majority of participants also identified the objects with their assigned Group-name.

## 4. General discussion

The aim of this study was to assess if verbal context (names) can alter touch behaviour and affective perception of tactile object. We found—in two experiments—that affect linked to the objects name (Positive, Neutral and Negative) linearly related to time spent touching an object, and their perceived pleasantness and disgust. That is, positively named objects tended to be touched for the longest durations, rated as most pleasant and least disgusting. Negatively named objects tended to be touched for the shortest durations, rated as least unpleasant and most disgusting. While there was near-perfect recall of the objects in their second (unnamed) presentation, they had lower affective ratings (see [17,19] for similar habituation effects). While we did not examine if object properties (e.g. textural or thermal) influenced the verbal context effects reported here, this would be one important avenue for future research.

In the chemical senses, a participant's pleasantness rating of a given taste, smell or food tends to move towards the valence of the provided name. This is known as an *assimilation* verbal context effect [21,22]. The three determinants of assimilation in the chemical senses may also apply for touch. The first determinant is the perceiver's *expectation* of the object, driven by past-experience—i.e. does the name generate expectations of how the object should feel (e.g. its texture and shape), before contact? The second determinant concerns object *ambiguity*. Ambiguous objects can be more unfamiliar, and multi-featured (e.g. multi-textured/shaped), allowing the perceiver to draw attention to object features congruent with their expectations. Modality-specific properties may also favour greater perceptual ambiguity in some senses over others. As noted in the Introduction, odours are highly ambiguous as they are poorly localized, and difficult to verbally describe [16]. By contrast, humans can recognize an extensive number (approx. 100–200) of everyday objects via touch [23,24]. Thus, relative to olfaction, a smaller range of touch objects susceptible to verbal context effects may exist. The third determinant is *congruency* between the expected and actual feel of the object, which determines if (and the extent to which) assimilation occurs [22]. Work is now needed to test the interactive and individual role of these attentional and learning-based determinants in tactile perception.

In conclusion, there appears to be a lot to the name for a touch object. Our behavioural and self-report findings indicate that verbal context is a powerful determinant of tactile behaviour and affective perception.

## Data Availability

All data used in analyses as well as a 'read_me' file (indexing key variables), have been uploaded on to Dryad Digital Repository: https://doi.org/10.5061/dryad.zgmsbccfr [25].
